# Perceptions, barriers and enablers of salt reduction in Malaysian out-of-home sectors (MySaltOH): from the point of view of policy-makers and food industries

**DOI:** 10.1186/s12961-023-00965-z

**Published:** 2023-02-09

**Authors:** Zaliha Harun, Suzana Shahar, Yee Xing You, Zahara Abdul Manaf, Hazreen Abdul Majid, Yook Chin Chia, Hasnah Haron, Viola Michael, Noor Shahida Sukiman, Aida Farzana Mohamad Taib, Feng J. He, Mhairi K. Brown

**Affiliations:** 1 Dietetic Programme, Centre for Healthy Ageing and Wellness (H-Care), Faculty of Health Sciences, Universiti Kebangsaan Malaysia, Jalan Raja Muda Abdul Aziz, 50300 Kuala Lumpur, Malaysia; 2 Centre for Population Health, Department of Social and Preventive Medicine, Faculty of Medicine, University of Malaya, 50603 Kuala Lumpur, Malaysia; 3 Department of Medical Sciences, School of Medical and Life Sciences, Sunway University, 47500 Bandar Sunway, Selangor Darul Ehsan Malaysia; 4 Department of Primary Care Medicine, Faculty of Medicine, University of Malaya, 50603 Kuala Lumpur, Malaysia; 5 Nutritional Sciences Programme, Centre for Healthy Ageing and Wellness (H-Care), Faculty of Health Sciences, Universiti Kebangsaan Malaysia, Jalan Raja Muda Abdul Aziz, 50300 Kuala Lumpur, Malaysia; 6 Allied Health Sciences Division, Ministry of Health Malaysia, Level 2, Block A, Chancery Place Main Building, Jalan Diplomatik 2, Precinct Diplomatik, Precinct 15, 62050 Putrajaya, Malaysia; 7 Wolfson Institute of Population Health, Barts and The London School of Medicine and Dentistry, Queen Mary University of London, London, EC1M 6BQ United Kingdom; 8 School of Chiropractor, AECC University College, Parkwood Campus, Parkwood Road, Bournemouth, Dorset, BH5 2DF United Kingdom

**Keywords:** Perception, Barriers, Enablers, Salt reduction, Out-of-home sectors

## Abstract

**Background:**

High salt intake is a major cause of hypertension and cardiovascular diseases. The out-of-home sectors have been identified as one of the contributors of high salt intake in the population. The National Salt Reduction Policy of Malaysia was initiated in 2015; however, out-of-home sectors are yet to be emphasized and perception by policy-makers and the food industries towards salt reduction are yet unknown. This study aimed to determine the perceptions, barriers and enablers towards salt reduction in the out-of-home sector in Malaysia, as well as among policy-makers and the food industries.

**Methods:**

This is a qualitative study via semi-structured in-depth interviews (IDI) and focus group discussions (FGD) involving several stakeholders consisting of policy-makers from five ministries, five nongovernment organizations (NGOs) and food science/food technology researchers from five regions (West, North, East, and South Peninsular and East Malaysia/Borneo), as well as the food industries. The IDI and FGD sessions were recorded, transcribed verbatim and analysed thematically using Nvivo software version 12.

**Results:**

All participants agreed that salt intake in Malaysia is high and leads to hypertension and cardiovascular diseases. Lack of awareness, poor eating culture and behaviour and frequent eating out were among the causes of high salt intake. Awareness campaigns and education, sodium content labelling and product reformulation were strategies that have been implemented by the government; whilst for the food industries, some of them have tried to reduce salt and labelled the sodium content on their food products. However, there were several barriers including perceived poor consumer acceptance, lack of knowledge and resources, and challenges in reformulation, as well as unavailability of guidelines and salt targets. Hence, several enablers have been suggested, which include prioritizing the salt reduction strategy, creating more awareness, collaboration and engagement, research and technology particularly for reformulation and shelf-life stability, incentives and salt tax.

**Conclusions:**

Salt reduction efforts of the out-of-home sector in Malaysia could be achieved through several measures or enablers that can overcome the barriers currently faced by stakeholders, especially policy-makers, food industries and the consumers themselves. This study will benefit the policy-makers to improve the salt reduction policy of out-of-home sectors and highlight the concerns among the food industries on the policy.

## Background

Cardiovascular diseases (CVDs), mainly coronary artery disease and stroke, are the leading cause of death worldwide, accounting for 31% of total global deaths [1]. Malaysia is not exempted from this; in fact, the Department of Statistics Malaysia reported an increase in deaths due to cardiovascular disease (ischemic heart disease) from 15% in 2019 to 17% in 2020 [2]. Of all the risk factors, hypertension was reported as the main contributor to the CVDs, which affects 30% (approximately 6.4 million) of Malaysians aged 18 years and older [3]. The WHO estimated that 1.7 million annual deaths from CVDs were attributed to excess dietary sodium intake [4] and the Malaysian Community Salt Survey conducted in 2017–2018 showed that 79% of Malaysians take an excessive amount of salt [5]. On average, Malaysians consume about 7.9 g salt (or 3167 mg sodium) per day. which is above the WHO recommended salt intake of 5.0 g per day [5, 6].

Salt reduction is among the most cost-effective measures in the prevention of cardiovascular diseases [7]. The WHO has set a global target of 30% reduction of salt intake by 2025. This has encouraged many countries in the world to speed up their salt reduction efforts. The strategies include food reformulation, consumer education, front pack labelling and interventions in public institution settings. Bread was the most targeted food for reformulation, followed by bakery products, processed meats, dairy products, sauces and convenience meals [8]. More countries are now opting for structural or regulatory approaches, however, efforts must be urgently accelerated and replicated in many countries and more rigorous monitoring and evaluation of strategies is needed to achieve the salt reduction target [9]. Within the South East Asia regions, only a few countries have dedicated stand-alone salt reduction policies; most are embedded in their national health/nutritional policy or overall noncommunicable disease (NCD) policies [10].

Malaysia began its salt reduction initiative in 2010 when a salt reduction committee was formed under the noncommunicable disease (NCD) section, Disease Control Division, Ministry of Health (MOH). The MOH announced a reduction of salt in 11 food items including soy sauce. Furthermore, the salt reduction committee collaborated with several agencies to increase awareness of salt and promote salt reduction in Malaysia [5]. The salt reduction strategy for Malaysia in 2015–2020 aimed at achieving the voluntary global target reduction of 30% in the mean population intake of salt/sodium by the year 2025. The midterm evaluation of this Salt Reduction Strategy 2015–2020 by WHO showed that there was only moderate progress in terms of delivering outputs to the Malaysian population and poor progress in engaging food manufacturers to reformulate food with less salt. Additionally, progress was also slow on salt reduction messages and education materials, particularly on the need to lower processed food intake. Thus, a continuation of the national reduction strategy has been outlined for 2021–2025, with a long-term target of 6.0 g of salt intake per day by the year 2025. Six recommendations to strengthen the salt reduction strategy were proposed, one of which was to address salt levels in the out-of-home sector [11]. The out-of-home sectors could be divided into a formal sector that includes registered and licensed restaurants, cafes, fast food outlets and takeaways, while the informal sector includes street food vendors or hawkers and informal food deliveries [12].

According to Department of Statistics Malaysia, about 17.3% of household expenditure was on foods and nonalcoholic beverages, while about 13.9% was on restaurants and hotels [13]. This data indirectly show that the out-of-home sectors are among the sources of foods that have an impact on sodium intake in the population. Out-of-home food intake is popular as it provides immediate or ready to eat foods. Thus, there is a need to specifically outline strategies to reduce salt consumption in out-of-home food sectors. To develop these strategies, further understanding of the out-of-home food industries and manufacturers as well as that of policy makers are needed.

This study aimed to determine the perceptions, barriers and enablers of salt reduction in the out-of-home sectors among the policy-makers, nongovernmental organizations (NGOs) and researchers, as well as the food industry. This study is part of a larger study on “Developing a policy to reduce the salt content of food consumed outside the home in Malaysia,” involving all formal and informal sectors [14, 15].

## Methods

### Study design and ethics

This study was a qualitative study in which participants were asked to openly share their views on the study matter, including the perceptions, barriers and enablers of salt reduction in Malaysia, with emphasis on the out-of-home sectors through 19 sessions of semi-structured in-depth interviews (IDI) and three sessions of focus group discussions (FGD). Due to the COVID-19 pandemic throughout the study period (May 2020 to March 2022) [16], a mixed approach of online meetings via the Google Meet application and face to face were carried out throughout the study. This research involved targeted stakeholders that were identified as related to the salt reduction policy, i.e., policy-makers and food industries. Ethics approvals were obtained from Universiti Kebangsaan Malaysia Medical Research Ethics Committee (UKM PPI/111/8/JEP-2020–524), the Malaysian National Medical Research Ethics Committee [ NMRR-20-1387-55481 (IIR)] and Queen Mary (University of London) Research Ethics Committee (QMERC2020/37) prior to the data collection. Written informed consent was obtained from all participants. To ensure confidentiality, pseudonyms were used in all interviews and transcriptions of the data has been anonymized. Identifiable information of participants was only used for arranging the interviews and obtaining signed consent, while demographic data was compiled in a table. Records were stored securely on a password protected computer, while the consent form was stored separately in a locked cabinet.

### Study framework and questionnaire

Questionnaires used in this study was developed based on a combination of framework modified from the Medical Research Council (MRC), UK [17], Ecological Model [18] and Theoretical Domains Framework (TDF) [19], as described elsewhere [14]. The basic framework adopted the development and feasibility domain from the MRC, while the Ecological Model and TDF were used to specifically refine the development process consisting of specific domains to answer the research question. Two set of questionnaires were developed, pretested and piloted in this study (Appendix A). The first questionnaire targeted the policy-maker, while another set is tailored towards the food industry. The questionnaires were pretested and piloted separately in a group of policy maker (i.e., two representative of policy-makers) and food industry (i.e., three representatives of food industry). After the pretesting and pilot study, the questionnaires were paraphrased to make it clearer to both the interviewer and respondents in the subsequent data collection. Since the amendment only involved paraphrasing of a few sentences, not involving major change of the domain of the questionnaire, the respondents involved in the pilot study were included as the actual sample for data collection.

### Sampling procedure and data collection

#### Sampling

The samples of this study were policy-makers and the food industries. A policy-maker is defined as those who creates ideas and plans, especially those carried out by a government. The policy-maker involved in this study included representatives from the ministry/government agencies acting as the primary policy-maker. Other stakeholders usually involved in policy-making including researchers from nutrition, food science/food technology from universities, or government agencies and NGOs who indirectly play the role in supporting the salt reduction policy were also included. The list of policy-makers and food industries was obtained from various sources including the Technical Working Group for Salt Reduction Policy of the Ministry of Health (MoH), the Food Industry Association of Malaysia (FAM), government agencies such the Federal Agricultural Marketing Authority (FAMA), the Malaysian Fisheries Development Authority (LKIM) and the Malaysian Agriculture Research and Development Institute (MARDI).

In this study, the ministry/government agencies involved were representatives from four divisions of the Ministry of Health (MoH) namely Noncommunicable Disease Division, Food Division, Food Safety and Quality Division, Nutrition Division and representatives from the Ministry of Education (MoE), Ministry of Domestic Trade and Consumers Affairs and the Ministry of Agriculture (MoA) and its agencies, which were the Federal Agricultural Marketing Authority (FAMA), Malaysian Fisheries Development Authority (LKIM) and Malaysian Agriculture Research and Development Institute (MARDI). Inclusion criteria of representatives of each organization included an officer or person in charge or well versed with food or salt reduction strategy or policy for at least 6 months. Five nongovernmental organizations (NGOs)/professional societies that participated in this study included the Malaysian Dietitians’ Association, the Malaysian Association for the Study of Obesity, the Family Medicine Specialists’ Association of Malaysia, the Malaysian Society Nephrology and the Malaysian Society of Hypertension. The researchers involved in this study were from several universities in Peninsular Malaysia and MARDI. The representatives from ministries, government agencies and NGOs were mostly based in the west of Peninsular Malaysia and four government agencies were from the Borneo region. There were representatives of policy-makers from the government agency from each of the regions, in line with the samples of the food industries. The distribution of the participants according to stakeholders and regions are presented in Table 1. Invitation letters were sent through e-mail to all the participants and appointments with them were made before the interview and discussion sessions. Invitations were sent to 13 policy-makers, 6 NGOs and 25 food industries. The response rate was 77% for policy-makers (*n* = 10), 83% for NGOs (*n* = 5) and more than 100% food industries (*n* = 65). Total response rate was 91%. For policy-makers, there were some ministries/government agencies that were invited who sent more than one participant in the IDI session, hence for ten ministries/government agencies, there were a total of 15 participants attending the sessions. For NGOs, only one participant for each NGO attended the IDI session. For the food industries, a snowball technique was applied and huge participation was recorded as some of the industries attended were not in the list of the invitation but their participation was significant to reach saturation for the data on the food industry.

**Table 1:** Distribution of participants according to stakeholders and regions

Stakeholder	West of Peninsular Malaysia (WPM)	North of Peninsular Malaysia (NPM)	East of Peninsular Malaysia (EPM)	South of Peninsular Malaysia (SPM)	East Malaysia/Borneo (EM)	Sample size	Method of data collection
a. Policy-makers							
i. Government ii. Nongovernmental organization (NGOs) iii. Researcher	11 5 10	– – 3	– – 4	– – 1	4 – 2	15 5 20	IDI/FGD
b. Food industries	8	5	34	6	12	65	FGD
Total	34	8	38	7	18	105	

*IDI* in-depth interview, *FGD* focus group discussion

In-depth interviews (IDI) were conducted mostly online with policy-makers (ministry, government agency and researcher) using a specific questionnaire. The in-depth interview (IDI) was conducted by an interviewer (researcher) that been trained to conduct the IDI session using standard protocol and questionnaire. At each session, a moderator or interviewer will conduct the interview, with assistance from a co-moderator and a rapporteur. Prior to the IDI, the interviewer started with a short introduction and briefing. The interviewer asked the respondent to open their camera and ensured that their microphone was in a good condition. The interviewer let the respondent answer the questions based on his/her own views and thoughts. The interview lasted for approximately 1 hour, then the interviewer would wrap up the session with key points highlighted by the respondents or participants. Focus group discussions (FGD) were conducted face to face following a standard operating procedure (SOP), as outlined by the Ministry of Health (MOH) involving groups of researchers and the food industries.

#### Data analysis

All participants were assigned a code number and pseudonym. The code and pseudonym were used to identify quotes and comments made by the interviewees. The quotes and comments made by the interviewees were coded according to gender, stakeholder, region group. The policy-maker stakeholder was further divided according to the designated group (ministry/government agency or researcher or NGO) and numbered accordingly. The food industries were coded according to gender, food industries’ category and region, and numbered accordingly. The interviews and the discussions were recorded using a voice recorder. The recordings were verbatim transcribed after the session was completed. The transcripts were analysed thematically using Nvivo software (version 12; QSR International, Doncaster, Victoria, Australia). Electronic data including recordings of interviews and discussions as well as transcripts were stored in shared drives with specific password access within Universiti Kebangsaan Malaysia (UKM), and were only accessible to trained research team members.

## Results

As presented in Table 2, the mean age was 40 years old (range 21–74 years), with the majority being women (66%) and having received tertiary education (53%). Specifically, among the food industries, most had secondary education (57%). A quarter of the participants of the food industry were from snack food (25%) and processed food industries (22%).

**Table 2:** Sociodemographic data of the participants [presented as mean, *n* (%)]

Sociodemographic	Policy-makers (*n* = 40)			Food industries (*n* = 65)	All (*n* = 105)
	Government (*n* = 15)	NGO (*n* = 5)	Researcher (*n* = 20)		
Age (years)					
Range Mean ± SD Median	36–58 44 ± 7 43	37–74 55 ± 14 55	34–52 42 ± 6 40	21–70 38 ± 10 37	21–74 40 ± 10 39
Gender (*n*, %)					
Male Female	3 (20) 12 (80)	2 (40) 3 (60)	4 (20) 16 (80)	27 (42) 38 (58)	36 (34) 69 (66)
Education level					
Secondary Tertiary N/A	– 13 (87) 2 (13)	– 5 (100) –	– 16 (80) 4(20)	37 (57) 22 (34) 6 (9)	37 (35) 56 (53) 12 (12)
Food industries’ category (*n*, %)	–	–	–		
Snack food Processed food Bakery Sauce Beverage Frozen food Noodles Others (salt, seasoning, salted/pickled/fermented food, anchovy, healthy food, baby food, ice cream)				16 (25) 14 (22) 8 (12) 6 (9) 5 (8) 4 (6) 3 (5) 8 (12)	65 (100)

In this study, we identified several themes related to salt reduction in the out-of-home sectors in Malaysia, categorized under “Perception on salt intake and salt reduction policy in Malaysia,” “Barriers in salt reduction policy,” and “Motivation/enablers on salt reduction.”

### Perception on salt intake and salt reduction policy in Malaysia

Generally, all participants in this study agreed that the salt intake among Malaysians is high and leads to health problems. They have highlighted several factors that influenced Malaysians to take high salt foods/food products such as the use of various sauces, salted fish, anchovies and food seasoning in food preparation. In addition, they have also shared their thoughts on the salt reduction policy including positive perception on the policy to reduce salt intake and ineffectiveness/insufficiency of the current policy, as well as opinions on the implementation of the policy. Table 3 summarizes the perception of the salt intake and salt reduction policy in Malaysia.

**Table 3:** Perception of salt intake and salt reduction policy in Malaysia

Theme	Sub-theme	Example of quotes
Perception on salt intake in Malaysia	Salt intake among Malaysians is high and could lead to health problems including hypertension and cardiovascular diseases	*“…we are consuming much more salt than recommended by the Malaysian dietary guideline or even by WHO”. (Female, Policy Maker/NGO 1)* *“…Hypertension can lead to a lot of stroke problem…” (Male, Food Industry/Soy Sauce, WPM)*
	Fast foods, processed foods, snacks, fermented and local seasoning foods, salted foods, pickled foods, sauces, seasoning/food enhancer contributed to high salt intake	*“Fast foods are among the options….” (Female, Policy Maker/Researcher, EM)* *“…they buy a lot of these sausages, frozen food, processed food…” (Female, Policy Maker/Researcher 1, WPM)* *“…sambal (chili paste) with belacan (shrimp paste) cincalok (fermented shrimp), tempoyak (fermented durian) are high in salt…” (Female, Policy Maker/Ministry 2, WPM)* *“… all the outside hawkers been selling their salty keropok lekor (fish cracker), chicken fried… are all salty” (Male, Policy Maker/NGO 5/WPM)* *“… snack food is more of a culprit you know…” (Male, Food Industry/Soy Sauce, WPM)* *“…if no monosodium glutamate (MSG), not tasty…” (Female, Policy Maker/Researcher 4, EPM)* *“…sauces and dressing, the seasoning, the soy sauce, chili sauce, tomato sauce and the sweet soy sauce, salty soy sauce all those are the food products with the high sodium” (Female, Policy Maker/NGO 2, WPM)*
	Cultural, behavioral, sociodemographic, family factors, availability of food and eating outside influence salt influence salt intake	*“…our traditional foods are high in salt like kolok and Sarawak noodles, layer cakes…” (Male, Policy Maker/Researcher, EM)* *“…for kids, they like to eat rice with soy sauce… (Female, Policy Maker/Ministry 2, WPM)* *“…at home parents decide foods for the family…” (Female, Policy Maker/Ministry 8, WPM)* *“… in the rural areas, they will use more of these preserved or highly processed foods, compared to the urban areas.” (Female, Policy Maker/Researcher 1, WPM)* *“…They start to dish snack together with their rice…Because they are so poor…So that’s what snack goes into.” (Male, Industry/Salt, WPM)* *“…high salt consumption either from the eateries… the restaurant, the people who prepare food…” (Female, Policy Maker/NGO 3, WPM)* *“…24-h restaurants/stalls/street foods are everywhere, and the availability and affordability…” (Female, Policy Maker/NGO 2, WPM)*
Perception salt reduction policy	Salt reduction policy is led by the Ministry of Health (MOH) as the ministry have the most authority on the policy	*“…the main source is MOH… but the delivery is through teachers, community, NGOs…” (Female, Policy Maker/Ministry 6, WPM)* *“…we don’t have the authority but we can perhaps create an impact…” (Female, Policy Maker/Researcher 1, WPM)*
	Salt reduction is feasible	*“Reducing the salt intake for the population is feasible. There are actually so many people out there who do want to eat less salt…” (Female, Policy Maker/Ministry 4, WPM)*
	Not very effective and insufficient	*“…our current strategy is insufficient… We need to admit our current strategies are not effective.” (Male, Policy Maker/Ministry 5, WPM)*
	Salt reduction policy should start off with voluntary and gradually reduce the salt intake before mandatory policy could be implemented	*“…we need to get at least one or two companies to show that they can do it… then perhaps voluntarily we will get some other companies to join…” (Female, Policy Maker/NGO 1, WPM)* *“… if we change gradually…so they can accept the new formulation…” (Male, Food Industry/Processed Food, NPM)* *“So, when the government has made it mandatory, maybe the reduction will gradually happen because of the mandatory order…” (Female, Food Industry Local Fast-Food, NPM)*

The current salt reduction policy by the Ministry of Health (MOH) includes guidelines to reduce salt in food products, sodium labelling, reformulation of foods, training and awareness campaigns as well as recognition and incentives that attract the food industries to reduce the salt content in their food products. Table 4 lists some of the salt reduction policies by the MOH and the practices of the food industries with regards to the policy.

**Table 4:** Salt reduction policy and industrial practice

Sub-theme	Policy-makers		Food industries	
	Salt reduction policy	Example of quotes	Industrial practice	Example of quotes
Reducing salt in food and food products	The MOH has promoted the salt reduction in the Malaysian Dietary Guideline	*“…under the Malaysian Dietary Guideline Message No. 9 there is about less salt… (Female, Policy Maker/Ministry 3, WPM)*	There are food industries that use salt substitutes/alternatives and technologies to reduce salt in the processed foods. However, there were some food industries do not have initiative to reduce the salt in their food products	*“So far, we haven’t done reduction…”(Female, Food Industry/Soy sauce, EPM)* *“…replace part of the sodium with potassium…” (Male, Food Industry/Sauce, WPM)* *“…If I use anchovy, I will not add salt… (Female, Food Industry/Processed Food 1, EPM)* *“…I use retort…for this technology, we don’t have to use preservative…” (Female, Food Industry/Processed Food 2, EM)*
Sodium labelling	The mandatory salt labelling has been gazetted in 2020	*“…in July 2020 we have gazetted amendment on food regulation to make a declaration of sodium a mandatory.” (Female, Policy Maker/Ministry 3, WPM)*	Sodium is being labelled on most processed foods, frozen foods, baby foods, beverages and fast foods but not on food products that being sold in bulk	*“…we make according to US standard, so need to have sodium content…” (Male, Food Industry/Beverage, NPM)* *“…we sell in bulk… so we don’t label because our reseller will repack…” (Male, Food Industry/Wholes Seller Snacks, WPM)*
Food reformulation	The government has engaged the food industries to reformulate the food products	*“…actually in 2018… Amendment of the salt regulation on soy sauce from 10 to 7% was gazetted” (Female, Policy Maker/Ministry 3, WPM)*	There were also food industries that have reformulated their food product to reduce the salt content	*“… for 25 kg flour, I used 1.5 kg salt. Then I did R&D to reduce the salt. Now I just use 400 g…” (Male, Food Industry/Noodle, EM)*
Training	The MOH have conducted Health Catering Training to teach the cooks on salt reduction in cooking	*“…we have been conducting ‘Health Catering’ training…We teach the food operators to reduce salt in foods…” (Female, Policy Maker/Ministry 2, WPM)*	The food industries have attended the training conducted by the government to use salt in appropriate amount	*“…when I attended course by FAMA, the officer said that I can add more salt to increase shelf life, but don’t follow my preference…as more salts would increase saltiness and increase cost too” (Female, Food Industry/Processed Food 2, EPM)*
Awareness campaign	The MOH together with the non-governmental organizations have conducted the salt reduction campaign	*“…we work closely with the Ministry of Health in their campaign to reduce salt intake…” (Female, Policy Maker/NGO 1, WPM)*		
Recognition and incentives	Healthier Choice Logo and MyChoice Logo were given to healthy foods while BESS-*Bersih* (clean), *Selamat* (safe) and *Sihat* (healthy) certificate were granted to premises that provide healthy foods	*“.…one of the criteria in the Healthier Choice Logo for certain food is the salt (sodium) …” (Female, Policy Maker/Ministry 2, WPM)* *“… MyChoice Logo, the requirements are reduced sugar, reduced salt… (Female, Policy Maker/Researcher 7, WPM)* *“… we have a certification called BESS…” (Female, Policy Maker/Ministry 3, WPM)*		

### Barriers and enablers of the salt reduction policy

There were several barriers, motivations enablers to salt reduction that have been proposed by the policy-makers and food industry participants. The barriers and motivation/enablers’ themes were divided into several categories including awareness, behaviour, consumer acceptance, knowledge, reformulation, resources, guidelines and salt target, priority, support, monitoring and law enforcement, price and cooking (Table 5).

**Table 5:** Barrier and motivation/enablers of the salt reduction policy

Sub-theme	Theme		Example of quotes
	Barriers	Motivation/enablers	
Awareness	Lack of awareness to reduce daily salt intake	Awareness to healthy life and prevent salt-related diseases	*“…Malaysians do not have awareness on salt reduction…”(Male, Food Industry/Snacks, WPM)* *“…we going to reduce all the cardiovascular complications and morbidities and mortalities if you prepare good food with less salt” (Female, Policy Maker/NGO1, WPM)*
Behavior	Exposure of high salt since young and bad eating habit	Creating awareness to change behavior on taking less salt needed to start from young	*“…when we were exposed too much…it became our norm…” (Female, Researcher 2, Central region)* *“…because of our habit is since young…”(Female, Policy Maker/Ministry 4, WPM)*
Consumer acceptance	The consumer complaint about the taste of the reduced salt foods	Continuously creating awareness to the public to increase their acceptance on lower salt products	*“…if we reduce the sodium, the consumers less prefer it…” (Female, Food Industry/Sauce, NPM)* *“…he brought this idea of having low sodium salt in this country where he can mix potassium chloride and sodium chloride…one the society will aware…” (Male, Seasoning Industry, Central region)*
Knowledge	Lack of knowledge on daily recommended salt intake and salt labelling	Increase of knowledge on salt reduction and salt labelling through promotion via mass and electronic media	*“…some people might not even know the daily recommended salt intake…” (Female, Policy Maker/Researcher 4, EPM)* *“… our consumer might not be ready yet to understand how to use this label.” (Female, Policy Maker/Researcher 1, WPM)* *“…use social media…” (Male, Policy Maker/Researcher, SPM)* *“…through their tv programs, radio…get up blast, non-stop…” (Male, Policy Maker/NGO 5, WPM)*
	Lack of knowledge on salt substitute	Educate the food industries how to use salt substitute	*“…is the salt substitute available in the market?… Does it change or have any effect?” (Male, Food Industry/Processed Food, NPM)* *“…What are the applications that are suitable and are not suitable…if they are not strong in their research, they wouldn’t know how to use it…” (Male, Food Industry/Salt, WPM)*
	Lack of knowledge to reduce salt in cooking	Educate reduction of salt in cooking with the use of natural ingredients, herbs and spices	*“…cut down your soy sauce and sauces… whatever you fry, don't add any salt…” (Male, Policy Maker/NGO 5, WPM)* *“…spices could alter the taste so that we can reduce salt…lemongrass and torch ginger can reduce the salt consumption…” (Female, Food Industry/Frozen Food, EM)* *“…use a lot of star anise and these stuff to cook meat products…. (Male, Food Industry/Sauce, WPM)*
Reformulation	Reformulation challenge using potassium chloride as it has an aftertaste and the problem of food stability and safety with the reduction of salt	Asist the food industries to do reformulation and introduce proper technology to increase the shelf life of the reduced salt products	*“…but potassium chloride got after taste… …” (Male, Policy Maker/Researcher, NPM)* *“…the shelf life will shorten…” (Female, Policy Maker/Researcher 4, WPM)* *“…the product cannot form properly…” (Male, Policy Maker/Researcher, EM)* *“…need help in terms of recalibrating or relining back their product reformulations…” (Male, Policy Maker/Government Agency 2, WPM)* *“…explore other methods how can we preserve food…”(Female, Policy Maker/Researcher 1, WPM)*
Resources	Lack of funding to do research, opting technologies in processing, production and marketing of lower salt products	Financial aid to do research, reformulation, using technologies in food processing, production and marketing of lower salt products	*“…very hard to get funding in research.” (Male, Researcher, East region)* *“… we are small and our cost of production is very high…” (Male, Food Industry/Sauce, WPM)* *“…for all the technologies, the cost is high…”(Female, Policy Maker/Research 4, WPM)* *“… resources are very limited…for marketing, product development…” (Female, Policy Maker/Ministry 2, WPM)* *“…to do all the reformulations, innovations…Need to put in a lot of money…” (Female, Policy Maker/Ministry 4, WPM)*
	Lack of funding and manpower could hamper campaigns	Grant is needed to run bigger salt reduction campaign	*“…I think the motivation is also definitely if we get some grants or something… if we are looking into doing a bigger or larger scale of awareness program, yes then funding might be needed….” (Female, Policy Maker/NGO 1, WPM)*
Guideline and salt target	No guideline and salt target make the food industries/providers to use excessive salt and the amount of salt used might vary between different manufacturers	Guidelines and salt targets are important especially for food industries/providers to reduce salt in their food products. There is in need of revision of the Food Act for the new salt target	*“…do not have the standard to determine whether the product is high in salt…no guideline…” (Female, Policy Maker/Government Agency 1, EM)* *“…if we don’t set the proper indicator of what we really want to monitor, what’s the impact we want to monitor. Then, could lose the target…” (Female, Policy Maker/Researcher 1, WPM)* *“…Revise the food act for the industry to follow…” (Female, Policy Maker/Researcher 1, WPM)*
Priority	Salt reduction is not a priority at the ministry and government agency level	Political involvement on salt reduction would make the issue become a priority for the government	*“…it should be prioritized when it comes to communication strategies…” (Male, Policy Maker/Ministry 1, WPM)* *“…so far, salt hasn’t been priority…” (Male, Policy Maker/Researcher, EM)* *“Perhaps when it involves politicians… to make it sound more serious and more doable” (Female, Policy Maker/NGO1, WPM)*
Support	Lack of support from other ministries, the food industries/providers and consumer on salt reduction	All stakeholders need to collaborate and support the salt reduction policy	*“…other ministries have their own policy and it is not aligned with our policy …” (Female, Policy Maker/Ministry 2, WPM)* *“…if the customers don’t want or have no demand…” (Male, Policy Maker/NGO 5, WPM)* *“…Bigger companies do have their own formulations… for us to introduce them with healthier options, it is quite tough…” (Female, Policy Maker/Researcher 2, NPM)* *“…Collaboration with all stakeholders…Starting from government, related authorities, researcher, school, parents, food industry…” (Female, Policy Maker/Researcher 4, EPM)*
Monitoring and low enforcement	Lack of monitoring and no law enforcement could drive the food industries away from the salt reduction policy as they feel safe from any punishment for not supporting the policy	Monitoring the salt content and low enforcement such as punishment or withdrawal of business license is needed to make sure the food industries/providers follow the requirement of lower salt content	*“…if there is no monitoring, no sampling and warning…maybe they will think there is no problem…no action will be taken unto them… So, need enforcement, implementation of act…” (Female, Policy Maker/Researcher, EM)* *“…if they are reluctant… then we have no choice to either boycott or give some form of a punishment or summon…” (Female, Policy Maker/NGO 1, WPM)* *“…the authority needs to take proactive action for example for hawkers that want to renew (license) they need to show that their foods are following the recommendation…” (Male, Policy Maker/Researcher 1, NPM)*
Price	Cheap price of salt, high salt price of lower salt/premium foods and low availability of lower salt products in the market halt the salt reduction policy	Increase salt price and impose taxation on salt and high salt food products	*“…products under the healthy category weather it is organic or natural, less salt… the price is premium… (Male, Policy Maker/Government Agency 2, WPM)* *“…It’s not easy to choose food that is low in salt. No choice…” (Male, Policy Maker/Researcher, EM)* *“…salt is the cheapest… low sodium salt would be more expensive…” (Male, Food Industry/Salt, WPM)* *“…they should be taxed…” (Female, Policy Maker/Researcher 1, WPM)* *“…raise the price of high salt foods…” (Female, Policy Maker/NGO 2, WPM)*

## Discussion

Our study showed that most of the participants agreed that the salt intake among Malaysians is high, as many food products including fast foods, snacks, processed foods, fermented foods and traditional foods, as well as food in the out-of-home sectors are high in salt content. The top ten food items that contributed to the highest sodium consumption among adult Malaysians include *kolok mee*, light soy sauce, curry noodle, vegetable with soy sauce/oyster sauce, fried instant noodle, noodle soup, vegetable with salted fish, fried vegetable, *roti canai/roti telur* and fried rice [20]. Findings by the Singapore National Nutrition Survey 2018 showed that the use of seasoning, sauces and salt in food preparation make up three-quarters of salt consumed in the diet [21]. Several of these foods are mentioned in this study by the participants and could be easily purchased as out-of-home foods.

Most of the participants in this study were aware of the association between high salt intake and health problems such as hypertension and cardiovascular diseases, and showed a positive perception towards the salt reduction policy to reduce the burden of these diseases. Continuous education and increased awareness among the population on salt reduction through electronic and mass media have been suggested by most of the participants. They also highlighted that very few campaigns/education pertaining to salt reduction could be found on television and social media as compared with other food components such as sugar. Other countries, such as the UK, reported similar findings [22]. Creating awareness among the consumers, food industries and health providers has been suggested as the first step in the salt reduction initiative as it will increase understanding on choosing foods with less salt, provide knowledge on the recommended daily salt intake and methods on preparing food with less salt [23]. However, since 2014, there has been a fall in consumer education around the world. This could be partially due to the fact that this strategy is resource and time-intensive, thus making it difficult to maintain [9]. The Ministry of Health of Malaysia [24] has also shared a similar view. There are calls for others such as nongovernmental organizations (NGO) to assist salt reduction advocacy, similar to what has been done by the Malaysian Society for World Action on Salt, Sugar and Health (MyWASSH), as endorsed by the World Hypertension League and the International Society of Hypertension [25]. Other NGOs that participated in this study did not indicate a specific plan in line with salt reduction strategies and policies.

Food labelling is one of the most important communication tools that can give information about the nutrient content of a food product, and hence can increase the awareness and knowledge on the salt content of an actual food product. However, not all food industry participants were aware of the announcement by the Ministry of Health for mandatory sodium labelling by July 2020, to be enforced by the year 2024 (26). The Healthier Choice logo has been introduced in Malaysia as a voluntary labelling, and encourages the food industries to produce healthy products since April 2017 [27]. However, its effectiveness on reducing the salt intake of the population has not been determined. There is a need to further explore perception of food industries on food labelling, as a recent study among Czech producers indicated that although there is a positive effect of food labelling on marketing, however, the effect on economic gain is not much [28].

Funding specifically for research and reformulation of lower salt foods was also emphasized as needed by most of the participants to produce lower salt foods. Several foods such as bread, meat, dairy and convenience foods have been successfully reformulated and contributed to the reduction of salt intake among the population in the UK [29]. Malaysia has reformulated about 53 food product categories including instant noodles, flavored cakes, sauces, biscuits, snacks and frozen meats, with a reduction of about 2–80% of their salt content. However, only a few of these reformulated products are high contributors of salt in diets, which makes a limited impact on overall intake of salt in the population [24].

Evidence from the UK and other countries has shown that reducing salt is relatively easy for manufacturers as it does not affect the products’ weight or volume and there are few technical barriers. Only for a small number of products does salt have a technical function. However, the large variations in the salt content of similar products clearly show how much salt can still be innocuously removed. Furthermore, consumers do not perceive any change in taste as salt is reduced gradually.

Salt is a poor preservative and is now rarely used as a preservative as other chemicals are more effective. There are also other technologies such as high-pressure, ultrasound, microwave and vacuum curing which have been adopted in the production of low-sodium fermented products and for the reduction of microbial contamination, acceleration of the fermentation process, improvement of texture and color, reduction of amount of salt added and shortening of the salting periods [30–34]. Improving the physical form of sodium salt to increase its dissolution and diffusion rate is one of the physical strategies that is also used in fermented foods [35].

The use of a salt substitute such as potassium chloride (KCl), calcium chloride (CaCl_2_), magnesium chloride (MgCl_2_), potassium lactate, calcium lactate and calcium ascorbate shows no risk of spoilage and pathogenic bacteria. However, these salt alternatives may produce a bitter taste, thus they could not completely replace the NaCl [36]. On the other hand, the use of salt synergists such ethanol and spices also have been utilized to increase the flavor and physicochemical properties of low salt products [37]. The use of functional microorganisms, microbial metabolites and enzymes could also improve the products’ quality and safety [38, 39]. Nevertheless, most of the respondents from the industries in the present study were not aware of techniques to reduce salt in their products without jeopardizing the shelf life and consumer acceptance.

Monosodium glutamate (MSG), which is widely used in cooking as food enhancer, has been suggested to increase the sodium content; however, several studies have reported that the addition of MSG in reduced salt foods could lower the sodium content in the foods. MSG contains two-thirds less sodium than NaCl and the saltiness is reportedly associated with a G-protein coupled receptor that enhances the perception of saltines [40, 41]. The permittable amount of MSG as a food additive in the range of 0.1–0.8% is equivalent to the amount of free l-glutamate found naturally in tomatoes and parmesan cheese [42]. Hence, we propose that the use of MSG needs to properly follow the recommendations for avoiding excessive use that leads to increased sodium content in foods. Our recent market survey indicated that processed foods with MSG added were among the foods with high sodium content [43].

The food industries also found difficulties in reducing the salt as they needed to follow the unrevised Food Act, which requires a fairly high amount of salt to achieve safety of food products. Hence, the current Food Act needs to be revised for a new salt target so the food industries can make the reformulations. Salt targets have been shown to effectively reduce salt intake in UK salt reduction strategy [44]. Many countries have shifted to approaches of salt reduction that change the food environment, such as providing lower salt options to the population and opting for regulation to support the change, such as through mandatory targets for salt levels in foods, mandatory front-of-pack labelling, mandatory nutrition standards in settings and salt taxation [9]. There were suggestions by policy-makers in this study on salt tax and subsidies for low salt foods. Salt tax has been implemented in various countries such as Fiji, Hungary, Mexico, Saint Vincent and the Grenadines, and Tonga on high salt foods and prepacked foods that exceed the recommended salt limit. In 2019, Thailand proposed a salt tax on high salt food including frozen products, canned foods and instant noodles [9]. Imposition of tax on high salt foods and subsidies for low salt foods could potentially result in a consumer choice for lower salt foods because of cost savings compared with their usual normal consumption [45]. In the current scenario of global increases in food price and inflammation, this strategy should be evaluated further.

The study has successfully obtained the perception, barriers and enablers towards a salt reduction strategy among policy-makers and the food industry. This qualitative study suggests the following: (i) Enhancing communication between policy-makers and the food industries to ensure shared responsibility in salt reduction strategies, (ii) introducing a visible front-of-pack label on food high in salt content, (iii) setting targets for voluntary salt reduction of a selective category of food high in salts, (iv) low-cost advocacy and education towards a lower salt consumption involving specific settings and using existing infrastructures such as schools, workplaces and community centres, and (v) monetary support for industries and researchers in the effort to reduce salt levels in salty foods.

However, there are several limitations, including that some of the policy-makers at the state level were not involved, and the online sessions conducted during the COVID-19 pandemic could have been better conducted face to face to obtain a better insight from the participants. Further research could focus on the effectiveness of current strategies such as Healthy Choice logos, toward a salt reduction strategy, and also innovative techniques to reduce salt in salty foods commonly consumed by the population.

## Conclusions

Perceptions on the salt reduction policy in Malaysia among policy-makers and the food industries were positive as they believe the policy could reduce the salt intake among the population and prevent noncommunicable diseases, particularly hypertension and cardiovascular diseases. Several challenges in implementing the salt reduction policy including acceptance by consumers of reduced salt foods, lack of knowledge and behavioural changes exist and can be addressed by education and continuous awareness campaigns. An absence of standards and guidelines for reformulations poses a challenge to producing lower salt food products. Priority issues and lack of support are other barriers that could prevent the success of the salt reduction policy. Hence, it is important to set a salt reduction target, encourage researchers to venture into research on salt reduction and engage the food industries to start with voluntary reformulation to reduce salt content in foods. Financial support is needed for research and reformulation, product development, use of possible technologies in food processing and to conduct bigger salt reduction campaigns. In summary, the salt reduction policy needs to involve all parties. Collaboration among ministries, researchers, nongovernmental organizations and the food industry is vital to make sure the policy that has been outlined by the Ministry of Health could effectively reach the global salt reduction target.

## Data Availability

All data were treated as confidential and not publicly available but could be disclosed through the correspondence author on a reasonable request.

## Appendix A

Questionnaire for policy maker and food industries.

**Table:**

No.	Item	Questions	
		Policy makers	Food industries
1.	Societal and cultural norm and values	1.1. What is your opinion on salt intake in Malaysia? Is it a problem? (Why the intake is high?) 1.2. What is/are other risk factor(s) beside dietary factor that you are worry about? Do you involve in development of policy to overcome this problem?	1.1 What is your opinion on salt intake in Malaysia? Is it a problem? (What the intake is high?) 1.2 Do you think that high salt intake will affect the health of the population?
2.	Government/organization/industry practice and structure/action	2.1.Does the ministry/organization have conducted/executed the strategy/programme to reduce the salt intake among Malaysia population? (Does it effective?) 2.2.What are other measures that need to done? – What is the target/objective? What are the actions to the execution? (Probe: Food labelling, law enforcement) 2.3.Who hold the responsibility to lead the reduction of salt intake among the population (How other sectors aside from health sector can assist in acceleration of salt reduction? (Probe: other opportunity, food industry, ministry/other party) 2.4.Who needs to involve in decision making/accelerate the changes? 2.5.Does salt target need to be mandatory? (How we can make sure industry/population to reduce the salt?) 2.6.What is the expectation on the propose action? (Does it can give benefit to the population and society?) (Probe: Economy benefit, health benefit etc.) Probe: Reformulation/modification/reduction of salt without populations aware. For example: United Kingdom do reformulation in bread processing-this action is voluntary without affecting the taste, quality and sale, the salt content reduces gradually every year 2.7.How the progress can be monitored/evaluated and who needs to do it? Is it independent body/NGO will assist?	2.1. Can you share about your product in the market? (Probe: what is your product that is low in salt?) 2.2 What is your opinion on customer/population acceptance towards low salt product? 2.3. Do your company try to reduce salt in their product? (Probe: Reformulation/modification/reduction of salt without populations aware. For example: United Kingdom do reformulation in bread processing-this action is voluntary without affecting the taste, quality and sale, the salt content reduces gradually every year. What is the support that you need to reduce salt in your product? 2.4 Do you practicing salt labelling on your product? 2.5 Do you add food enhancers other than salt in your product? What is the salt enhancer? 2.6. Do you use salt substitute in production of your product? If not, do you willing to do it? (E.g.: Potassium Chloride) 2.7. What is your opinion regarding salt reduction policy? Does this strategy need to be voluntary or mandatory? Why?
3.	Motivation	3.1. What is the factor that encourage the ministry/organization to participate/take action? 3.2. (If Question 2.2 have been answered: no need to answer Question 3.2; if related to NGO/ministry). Do the ministry/organization have long-term target to evaluate salt reduction commitment? (Probe: How do you do it?)	3.1. What is the factor that encourage you to participate//take action to reduce salt? 3.2. Do you expect the salt reduction strategy as part of the social responsibility to increase health among population? 3.3. If NO ACTION, go to no 4 3.4 Do you have a long-term target to evaluate salt reduction commitment? (Probe: How do you do it?)
4.	Barriers/Challenges	4.1. How do you face all the barriers to implement salt reduction strategy/policy? 4.2. Do you need support from other party to take action in you organization? (Probe: support; knowledge/ability/skills to take the action?)	4.1. How do you face all the barriers to implement salt reduction strategy/policy? 4.2. Do you need support from other party to take action in you organization? (Probe: support; knowledge/ability/skills to take the action?)
5	Monosodium Glutamate	5.1 What is your opinion on monosodium glutamate? (Probe: bad effect, purpose of using etc.)	5.1 What is your opinion on monosodium glutamate? (Probe: bad effect, purpose of using etc.) 5.2 Do you add in MSG in your product?
6.	Additional information	6.1 Is there any other issue/opinion that you would like to add?	6.1 Is there any other issue/opinion that you would like to add?
